# Radiation-Induced Lung Fibrosis: Preclinical Animal Models and Therapeutic Strategies

**DOI:** 10.3390/cancers12061561

**Published:** 2020-06-12

**Authors:** Hee Jin, Youngjo Yoo, Younghwa Kim, Yeijin Kim, Jaeho Cho, Yun-Sil Lee

**Affiliations:** 1 Graduate School of Pharmaceutical Sciences and College of Pharmacy, Ewha Womans University, Seoul 03760, Korea; hee_jin@ewha.ac.kr (H.J.); youngbird1210@ewhain.net (Y.Y.); qtechhwa@ewhain.net (Y.K.); kyeejin@ewhain.net (Y.K.); 2 Department of Radiation Oncology, Yonsei University Health System, Seoul 03722, Korea

**Keywords:** radiation, lung fibrosis, animal models, antifibrotic, therapeutic targets

## Abstract

Radiation-induced lung injury (RILI), including acute radiation pneumonitis and chronic radiation-induced lung fibrosis, is the most common side effect of radiation therapy. RILI is a complicated process that causes the accumulation, proliferation, and differentiation of fibroblasts and, finally, results in excessive extracellular matrix deposition. Currently, there are no approved treatment options for patients with radiation-induced pulmonary fibrosis (RIPF) partly due to the absence of effective targets. Current research advances include the development of small animal models reflecting modern radiotherapy, an understanding of the molecular basis of RIPF, and the identification of candidate drugs for prevention and treatment. Insights provided by this research have resulted in increased interest in disease progression and prognosis, the development of novel anti-fibrotic agents, and a more targeted approach to the treatment of RIPF.

## 1. Introduction

Radiation therapy (RT) is performed in about 50% of all cancer patients at least once during their treatment course. Recently, with the development of computer and mechanical engineering, it has become increasingly important through effective treatment. Clinical RT involves irradiation with high radiation doses to remove tumors. Surrounding normal tissue adjacent to the tumor is prone to side-effects. The most common side effects after ionizing radiation (IR) treatment of thoracic tumors are pneumonitis and pulmonary fibrosis (PF). While pneumonitis occurs early following treatment and may be reversible, PF is considered to be irreversible delayed toxicity [1]. Pneumonitis can occur in as many as 50% of lung cancer patients, and rates of PF can be as high as 70–80% in high-dose regions of the lung [2]. Although it affects fewer people than idiopathic pulmonary fibrosis (IPF) per se, similar pathological changes are seen in a minority of adult cancer survivors exposed to lung irradiation. Currently, there are no approved treatment options for patients with radiation-induced PF (RIPF), which may be mediated by the absence of effective targets. However, regimens for IPF, such as pirfenidone and nintedanib, help to reduce clinical exacerbations that affect pulmonary function [3,4]. In this review, to emphasize the importance of small animal models, we first described the characteristics of radiation-induced lung injury (RILI) including radiation pneumonitis and RIPF. In addition, we discussed the pathological mechanisms of RIPF including the cytokine secretion, and the epithelial and endothelial-to-mesenchymal (EMT/EndMT) process. Lastly, we also described the antifibrotic agents used in the clinic or under the investigations.

## 2. Radiation-Induced Lung Injury (RILI)

**Figure 1:**
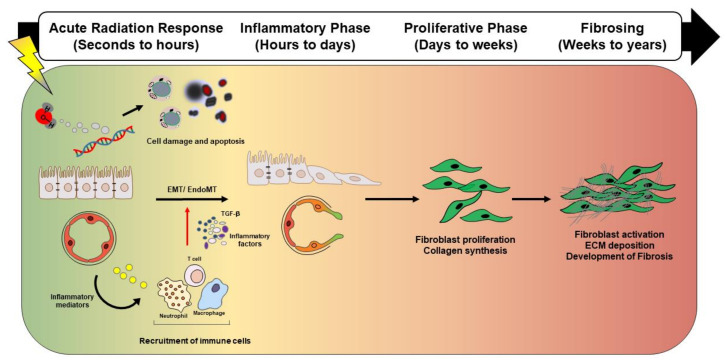
Schematic representation showing distinct and overlapping stages of radiation-induced lung injury. Radiation-induced lung injury consists of overlapping and highly coordinated stages of acute radiation response, inflammation, proliferation, and fibrosis. After lung injury, ionizing radiation induces reactive oxygen species (ROS) induction, DNA damage, and vascular damage. Damaged epithelial cells and/or endothelial cells release inflammatory mediators that recruit immune cells. The recruited immune cells secrete profibrotic cytokines such as IL-1β, TNF, IL-13, and TGF-β. Secreted cytokines amplify the inflammatory response and trigger fibroblast proliferation and recruitment, which eventually culminates in fibrotic changes. Abbreviation: ROS: Reactive oxygen species, IL-1β: Interleukin 1 beta, TNF: Tumor necrosis factor, IL-13: Interleukin 13 beta, TGF-β: Transforming growth factor-β, EMT: Epithelial to mesenchymal transition, EndMT: Endothelial to mesenchymal transition, ECM: Extracellular matrix

When IR passes through the lung tissue, energy not only directly induces double-strand break (DSBs) of the DNA molecule, but also has sufficient strength to hydrolyze water and other molecules. This hydrolysis produces reactive oxygen species (ROS), which can interact with DNA and other cellular components of extracellular matrix (ECM) [5]. Most DNA damage is repaired, but incorrect repair can lead to cell deficiency and apoptosis for a much longer time, and may even initiate a strong immune response even before tissue damage is induced [6]. IR exposure can lead to apoptosis of epithelial and endothelial cells within hours and this apoptosis has been experimentally demonstrated to occur in the lung parenchyma after injury [7]. Moreover, ROS can also be produced by cascades of pro-inflammatory cytokines, and the release of cytokines and other products of activated transcription factors play an important role in the progression of RILI [8] (Figure 1).

### 2.1. Radiation Pneumonitis

**Figure 2:**
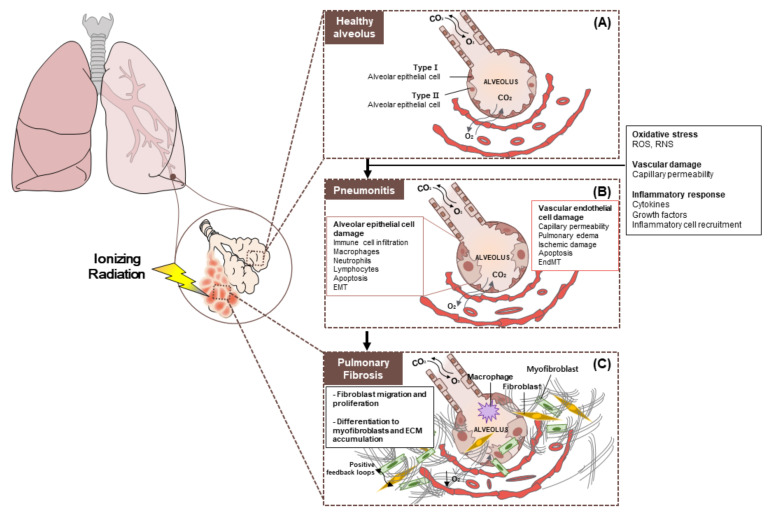
Pathobiology of radiation pneumonitis and radiation-induced lung injury. (**A**) Healthy alveolus. The epithelium, which is the outer layer of the alveoli, consists of two types of cells (type 1 and type 2 cells). Type 1 alveolar cells cover 95% of the alveolar surface and constitute an air-blood barrier. Type 2 alveolar cells are smaller and are responsible for the production of surfactants that coat the inside of each alveolus when inhaling and breathing. (**B**) Radiation pneumonitis is inflammation of the lungs caused by radiotherapy. Chronic pneumonitis can cause scarring of the lungs called pulmonary fibrosis. (**C**) Pulmonary fibrosis. General changes seen in interstitial lung disease. There is proliferation of interstitial fibroblasts and recruitment of fibrocytes into the interstitium (extracellular matrix (ECM)). These cells begin secreting components of the ECM such as collagen and other fibers. Cells also convert to myofibroblasts, which produce more ECM. Prolonged fibroblast activity leads to fibrosis, which is a hallmark of interstitial lung disease and restrictive lung patterns. Abbreviation: EMT: Epithelial to mesenchymal transition; EndMT: Endothelial to mesenchymal transition; ECM: Extracellular matrix

Radiation pneumonitis as an acute reaction occurs within 4–12 weeks after RT. The acute pneumonitis stage is characterized by recruiting various immune cells into the alveolar space, thickening the alveolar septum, and destroying the integrity of the alveoli. At this time, infiltration of myeloid and lymphoid cells occurs, which results in lung inflammation and edema of alveolar interstitium and the air space [9]. In the lung alveoli, which is the end of the respiratory tree, there are two types of alveolar epithelial cells (AECs or pneumocytes) known as AECI and AECII. In addition to epithelial cells, alveolar macrophages are present in the alveolar space, which are essential for tissue homeostasis, early pathogen recognition, and the initiation of local immune responses and resolution of inflammation. Resident fibroblasts are the most prominent cell type in the alveolar interstitium. Under certain pathological situations, resident fibroblasts can be activated and transformed into myofibroblasts. Myofibroblasts are the main effector cells of tissue fibrosis [10,11,12]. When ACEI undergoes apoptosis or necrosis, depletion of the basement membrane occurs, which is followed by hyperplasia of ACEII. However, the accelerated proliferation of ACEII inhibits the ability of lung surfactants production, which causes surface tension to be lost. This is followed by edema and lethargy of lung tissue [13]. Edema increases vascular permeability, which causes plasma proteins and fibrin-rich exudate to leak into the alveolar space. Radiation pneumonitis without specific treatment remains a major dose-limiting complication in patients. The current treatments including steroids, diuretics, hormones, enzymes, and antioxidants are still non-specific and symptomatic. If pulmonary damage is so extensive that homeostasis cannot be restored by any repair mechanisms, the integrity of the alveolar-capillary barrier begins to decompose at certain points where it does not recover. This makes RIPF worse due to inadequate regeneration and subsequent processing of lung tissue (Figure 2).

### 2.2. Radiation-Induced Pulmonary Fibrosis

RIPF occurs at an irreversible stage more than six months after irradiation. The characteristic RIPF is the accumulation of fibroblasts and myofibroblasts, causing extensive production of collagen, infiltration of inflammatory cells, and remodeling of ECM. This is followed by fibrosis of the alveolar septum, which, in turn, leads to extensive occlusion of the alveoli. RIPF is characterized by the accumulation of the ECM protein, which lowers the ability of the lungs to exchange oxygen. In response to IR, resident lung fibroblasts can differentiate into myofibroblasts, which can secrete ECM proteins. Activated myofibroblasts contribute to fibrogenesis. Therefore, inhibiting IR-induced myofibroblast differentiation is an important therapeutic strategy for preventing fibrosis [14,15]. IR activates fibroblasts/myofibroblasts, which leads to incorrect regulation and exaggerated repair processes, and eventually fibrosis [16]. Epithelial-mesenchymal transition (EMT) is one of the processes that causes lung fibrosis, which is activated by IR, but may also be involved in other pulmonary processes such as embryonic development [17] and tumorigenesis [18]. The major cytokine involving the EMT process is transforming growth factor-β (TGF-β). TGF-β signaling initiates the trans-differentiation of epithelial cells into activated myofibroblasts [17]. The main role of cells undergoing EMT is the synthesis and deposition of ECM proteins.

## 3. Animal Models of RIPF

### 3.1. Animal Models That Mimic Conventional RT

**Figure 3:**
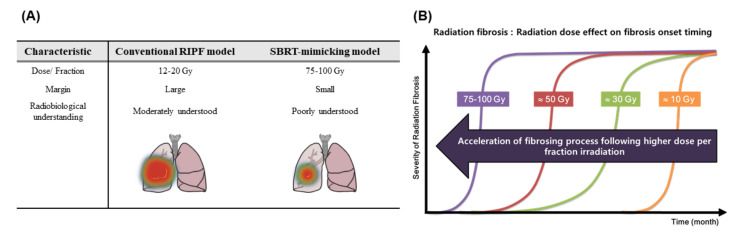
General comparison of conventional and stereotactic SBRT-mimicking RIPF models. (**A**) Summary of conventional and SBRT-mimicking RIPF models. In the conventional RIPF model, a large volume of the lung is irradiated with a relatively low dose of radiation, and an abundance of radiobiological research data has been generated. In contrast, the SBRT-mimicking RIPF model mimics modern radiotherapy whereby there is local irradiation with high radiation doses. (**B**) Both the dose and fractionation of radiation contribute to the severity of radiation fibrosis. RIPF is known to be accelerated with a higher radiation dose per fraction. Therefore, the radiation dose is very important for the development of RIPF. Abbreviations: SBRT: Stereotactic body radiation therapy; RIPF: Radiation-induced pulmonary fibrosis

Classical studies using whole thorax irradiation have provided important information about the pulmonary IR responses of different rodent strains and defined dose thresholds for lung toxicities. Susceptibility to fibrosis to IR is genetically based in mice, and, most notably, C3H/HeJ and CBA/J mice are resistant to IR-induced fibrosis in comparison with the more susceptible C57BL/6 strain [19]. C3H/He mice are used as a classical model to develop early-stage pneumonitis, but do not develop fibrosis below a single dose of 20 Gy by 12–20 weeks. In contrast, 20 Gy in C57BL/6 mice are sufficient to induce fibrosis, while other strains are intermediate between these extremes [20]. To avoid a systemic complication, RT that usually delivers thorax-limited IR is applied and, in this case, fibrosis takes up to 24 weeks [21,22]. During the pulmonary fibrosis, inflammatory responses affect the severity of lung remodeling but is rarely associated with mortality [19]. The pathology of RIPF is believed to involve ROS-mediated DNA damage and TGF-β production [23]. The introduction of the small animal irradiator significantly improves the precision and accuracy of the dose delivery to the mice, so that more clinically relevant dose and fractionation schedules could be experimentally achieved. When using a small animal irradiator targeting a 10% to 30% sub-volume of the lungs or the near whole lung, representative examples of the dose distribution is shown in Figure 3. Recently, only a small number of published studies focused on RIPF have aimed to improve understanding of the major biological and physical factors supporting the lung’s IR response as preclinical technology advances using a small animal irradiator. This suggests that, as the preclinical techniques advance, there are many challenges and opportunities to understand the biological and physical factors of the lung’s IR response. Fibrosis developed in response to application of 20 Gy to the entire left lung of C57BL/6 mice, and a significant amount of lung fibrosis was observed at 12 months after IR. Collagen deposition was observed at three months after IR with peak induction at 12 months. Fibrosis-related genes identified revealed several differences between 20 Gy IR and 75–90 Gy SBRT (stereotactic body radiation therapy)-mimicking irradiation, which suggested different molecular mechanisms of a low dose whole lung irradiation and a high dose focal irradiation in developing lung fibrosis [24].

### 3.2. Animal Models to Mimic Stereotactic Body Radiation Therapy (SBRT)

More sophisticated conformal RT techniques such as intensity-modulated RT (IMRT), volumetric arc RT (VMAT), stereotactic body radiation therapy (SBRT), and stereotactic radiosurgery (SRS) have a lower RILI incidence than standard 3D conformal RT. This can be due to the increased precision of IR delivery to the tumor, which, thereby, protects the surrounding normal tissue. Moreover, due to the diversity of the technology, it is possible to guide the intensity and direction of the radiation beam by taking into account the anatomy, breathing pattern, and organ motion of the patient. All of these modalities are expected to lower the volume of lung irradiation and the irradiation dose while maintaining millimeter-range accuracy. Moreover, SBRT is the standard of care for patients with medically inoperable stage I NSCLC (Non-small-cell lung carcinoma) [25]. The steep dose gradient and sharp dose fall-off achieved with SBRT substantially reduces treatment volume, and, thereby, reduces the dose to surrounding normal tissues. However, based on published data, the rate of symptomatic IR pneumonitis ranges from 0–49%. The rate of RILI, which can continue to evolve one year after SBRT, is still 70% to 80% in high-dose regions [26]. A mouse model simulating clinical SBRT in terms of ablative dose delivery in a few fractions has been used to show the induction of pulmonary fibrosis using image-guided high-dose-per-fraction irradiation system [27]. Fibrosis-related gene expression in the 90 Gy SBRT-mimicking model was different from that of the 20 Gy conventional RT model [24]. In the SBRT mimicking model, no difference in fibrosis progression between radiosensitive C57BL/6 and radioresistant C3H mice was detected [28]. Because fibrosis development was rapidly induced in this model (4–6 weeks) compared to the conventional RT animal model (6–12 months), it may be a good preclinical model to evaluate anti-fibrotic drugs.

## 4. Characteristics of RIPF

The pathological mechanism of RIPF is complex and involves numerous cell types. Thorax IR induces delayed damage to resident lung cells, which, primarily, leads to apoptosis of bronchial epithelial cells and loss of barrier function [29]. Recent studies have shown that damaged epithelial cells can become a source of myofibroblasts called EMT/EndMT (Epithelial and endothelial-to-mesenchymal transition) [30,31,32]. Myofibroblasts are known to play a central role in the pathogenesis of RIPF because these cells produce collagens, fibronectins, and other matrix molecules [33,34,35]. Moreover, thorax IR has been shown to induce recruitment of various immune cells into the lungs. After IR exposure, the expression of Th2-related cytokines increased, and the expression of Th1-related cytokines decreased. Thereafter, alveolar macrophage accumulation in the irradiated tissue increased, and TGF-β was dramatically expressed [36]. Macrophages can be divided into two subsets according to their distinct functions [37,38,39]. The two subsets are known as classically activated macrophages (M1 macrophages) and alternatively activated macrophages (M2 macrophages). M1 macrophages are known to play a primary role in the onset of injury [40,41,42]. They secrete pro-inflammatory cytokines that exacerbate damage, amplify the inflammatory response, and contribute to myofibroblast proliferation and recruitment of fibroblasts [43,44]. M1 macrophages are responsible for the release of matrix metalloproteinases (MMP) that break down ECM and promote EMT/EndMT [45], and M2 macrophages contribute to the control of inflammatory processes [46]. When the acute phase of inflammation is complete, Th2 cytokines are produced, which promote the polarization and recruitment of M2 macrophages [47]. In addition, M2 macrophages create an anti-inflammatory environment and promote wound healing [48] and regeneration [49]. However, in the lesion, M2 macrophages may play an important role in the pro-fibrotic process by secreting large amounts of pro-fibrotic factors [50] (Table 1).

### 4.1. Cytokine Responses in RIPF

Since various cytokines affect the particular processes of RIPF [51], cytokines have been intensively studied as signaling molecules related to the RILI process and as candidate biomarkers that can identify the risk of RIPF development. Although each cytokine has a unique expression profile, within 4–24 h after irradiation, there is often a nonspecific acute reaction (so-called ‘cytokine storm’) characterized by elevated expression of numerous cytokines. This is followed by a decrease in cytokine levels to baseline within a period of 24 h to a few days [52]. Hundreds of studies have examined the expression of cytokines after irradiation, and a wide spectrum of cytokine profiles are available for both patients and animal models. Damaged pneumocytes, resident macrophages, and endothelial cells release inflammatory cytokines such as TGF-β (Transforming growth factor-β), TNFα (Tumor necrosis factor-α), PDGF (platelet-derived growth factor), IL-1 (Interleukin-1) and IL-6 (Interleukin-6) as well as FGF (Fibroblast growth factor) [31,53,54,55,56]. The characteristic changes in the expression of local and systemic cytokines as well as chemokines are associated with the recruitment of immune cells [57]. Additional studies are necessary to define how the various cells interact and are coordinated during irradiation-induced pulmonary injury (Table 2).

### 4.2. EMT (Epithelial-Mesenchymal Transition) in RIPF

IR induced a series of complex injuries mediated by oxidative stress, cell death, senescence, and loss of normal lung barrier functions [75]. Some alveolar epithelial cells may undergo EMT and EMT is controlled by transcription factors such as Snail and Twist. Activation of these transcription factors inhibits E-cadherin and increases the expression of contractile protein α-SMA (Smooth muscle actin) [76,77,78,79]. The EMT associated with wound healing, tissue regeneration, and organ fibrosis is considered a second type of EMT. Type 2 EMT generally initiates the production of fibroblasts and other related cells to reconstruct tissues after trauma and inflammatory injury [80]. The production of numerous molecules by inflammatory cells and resident myofibroblasts is associated with Type 2 EMT. These molecules destroy the epithelial layer through the breakdown of the basement membrane. The loss of epithelial cells polarity induces apoptosis or EMT [17]. However, unlike to type 1 EMT, type 2 EMT is associated with inflammation and stops when inflammation is weakened during processes of wound healing and tissue regeneration. The combination of sustained myofibroblast activation, collagen deposition, EMT, and sustained activation of inflammatory cytokine signaling is likely to lead to fibrosis and collagen produced primarily by fibroblasts. This collagen is the most abundant matrix in fibrotic lesions.

## 5. Therapeutic Targets and Their Inhibitors in RIPF

### 5.1. Inflammatory Cells

The therapeutic strategy for treating lung injury after IR is restoring the immunological balance. With this treatment strategy, corticosteroids play a major role in managing acute IR pneumonitis, but its role in advanced lung fibrosis remains unclear. Inhibition of neutrophil elastase (NE) prevents the development of lung fibrosis after acute lung injury [81]. Irradiated mice that received sivelestat survived longer than mice that received IR alone. Sivelestat reduced RIPF in mice by suppressing NE activity and an excessive inflammatory reaction [82]. CSF1/CSF1R (colony-stimulating factor receptor-1) pathway is a novel therapeutic candidate for RIPF inhibition based on the results from a preclinical mouse model following thorax IR and also from using human lung biopsies from patients who underwent thorax irradiation. The murine counterpart of the human CS4 antibody known as the anti-CSF1R antibody (CS7) is currently in clinical trials for treating prostate and breast cancer [83]. Specific depletion of infiltrating macrophages in RIPF with CSF1R mAb had an anti-fibrotic effect, and CSF1R was identified as a potent antifibrotic target [84]. Navitoclax (ABT-263), a B cell lymphoma 2/extra-large (Bcl-2/xl) inhibitor, and a newly identified senolytic drug can reverse RIPF even at the late stages of fibrosis [85]. This finding suggests that ABT-263 is a potentially effective treatment for RIPF.

### 5.2. ROS (Reactive Oxygen Species)

The thiophosphate, amifostine, is the only agent approved by the FDA as a clinical IR protector and its active metabolite acts as a radical scavenger [52] to protect against lung and soft tissue fibrosis. Mammary adenocarcinoma bearing rats that received fractionated RT and were treated with amifostine showed reduced lung damage with low plasma TGF-β levels. In addition, administration of amifostine before IR also reduced macrophage accumulation and TGF-β1 expression in lung tissue [86]. Superoxide dismutase (SOD) is a metalloprotease that protects against oxidative damage [36]. Recombinant SOD-TAT (Superoxide dismutase fusion of TAT) protein protected against RILI in mice. When irradiated mice were examined after pretreatment with SOD-TAT, improved growth rate and reduced lung hydroxyproline content as well as sustained SOD activity, glutathione peroxide (GSH-Px) activity, and total antioxidant capacity were detected [87]. 

### 5.3. Cytokines/Chemokines

Toll-like receptor (TLR) activation requires a number of intracellular adaptor proteins proximal to TLR and the most prominent one is a myeloid differentiation primary response factor 88 (MyD88). MyD88 contains two prominent domains, a TLR/IL1 receptor domain, and a death domain. MyD88 is a key control factor for innate immune signaling by pathogenic and environmental stresses. MyD88 regulates innate immunity, reduces long-term RILI, and alleviates fibrosis development by preventing chronic lung damage [29,88]. Activation of the chemokine receptor CXCR4 by its ligand stromal cell-derived factor 1 (SDF-1/CXCL12) may also involve the development of RIPF [89]. MSX-122 is a novel small molecule and partial inhibitor of CXCR4, and the CXCR4/CXCL12 axis has been shown to be critical for the development of RIPF in a mouse model. CXCR4 inhibition by MSX-122 may alleviate potential RILI, which represents a therapeutic opportunity for patients requiring chest irradiation [89].

### 5.4. TGF-β (Transforming Growth Factor-β)

Activation of the TGF-β/Smad signaling pathway, which is an important step for fibrosis development, has been explored as a potential intervention strategy. SM16, a small molecule inhibitor of the TGF-β receptor I, reduced the degree of RILI in a rat model [29]. Rats that received SM16 experienced a significant decrease in PF, inflammatory response, and TGF-β1 activity [90]. LY2109761 is a novel small-molecule TGF-β receptor I serine/threonine kinase inhibitor that markedly reduces inflammation and PF by IR, which results in prolonged survival. This inhibitor directly attenuates profibrotic signaling pathways as well as mediates TGF-β/bone morphogenetic protein (BMP) signaling [91]. Galunisertib is a highly selective inhibitor of TGF-β receptor I by complete inhibition of its phosphorylation and activation [92]. Treatment with galunisertib attenuated IR-induced pulmonary inflammation and fibrosis. SB203580 and WP631 inhibited the Smad signal transduction pathway, abrogated excessive proliferation, and reduced the expression of p21 (WAF1/CIP1) and PAI-1 (Plasminogen Activator inhibitor-1) induced by IR and TGF-β1 [29]. SB203580, which is a pyridinylimidazole, can highly and specifically inhibit TGF-β receptor I. WP631, which is a serine proteinase inhibitor, inhibited the expression of PAI-1. An increase in expression and activation of PAI-1 could effectively prevent hydrolysis of fibrous proteins and degradation of the ECM, which causes the precipitation of fibrous proteins within tissues and the accumulation of ECM [93]. Thalidomide was shown to inhibit TGF-β1-induced α-SMA and vimentin production as well as pulmonary epithelial cell morphological changes by suppressing both Smad-dependent and non-Smad-dependent pathways. Thus, thalidomide may have potential as a therapeutic agent to inhibit pulmonary fibrosis [89].

### 5.5. PDGF (Platelet-Derived Growth Factor)

The platelet-derived growth factor (PDGF) receptor tyrosine kinase inhibitors significantly attenuate the development of fibroblast foci, characteristics of PF, and subsequent remodeling of the pulmonary structure. SU9518 is a highly selective inhibitor of the PDGF receptors α and β, which blocks PDGF receptor kinase activity and PDGF receptor-induced cell proliferation [36]. SU9518, SU1167, and imatinib mesylate with all activity against PDGF receptor α and PDGF receptor β have been shown to attenuate PF in an animal model [94]. SU9518 treatment significantly reduced collagen deposition, alveolar wall thickness, and other histological signs of fibrosis [95,96].

### 5.6. CTGF (Connective Tissue Growth Factor)

Connective tissue growth factor (CTGF) is known as a molecule for TGF-β-mediated fibrosis. However, CTGF, once activated, has been reported to promote and maintain PF by exerting a direct fibrosis effect out of control by TGF-β. This hypothesis is supported by data demonstrating that CTGF activates collagen type 1 and promotes ECM synthesis. CTGF depletion in fibroblasts and smooth muscle cells significantly reduced fibrosis [97]. Pamrevlumab (FG-3019), which is a human monoclonal antibody against CTGF, exhibits reprogrammed fibrogenesis through normalization of IR-induced genes. These genes regulate inflammation associated with M2 macrophage influx, EMT, myofibroblast activation, tissue remodeling, and ECM deposition. Treatment of FG-3019 to irradiated mice reversed lung regeneration, preserved lung function, and increased survival [36,98,99].

### 5.7. Miscellaneous Targets

The renin-angiotensin system is known to regulate blood pressure and fluid balance and has also been reported to be involved in the development of RILI. Some angiotensin-converting enzyme (ACE) inhibitors have proven to protect the lungs from fibrosis after IR exposure [97]. Treatment of three structurally different ACE inhibitors (fosinopril, captopril, and enalapril) in drinking water after whole thorax IR reduced collagen synthesis [100]. Methoxystradiol (2-ME), which is a metabolite of 17-beta-estradiol, has been proposed to effectively inhibit HIF-1α. 2-ME suppressed the IR-induced increase of HIF-1α (Hypoxia-inducible factor 1 alpha), which led to a decreased EndMT. The deposition of vascular collagen during RIPF development was also inhibited [33,36]. Myriocin is a specific inhibitor of serine palmitoyltransferase (SPT) and can block the de novo biosynthesis of sphingolipids. Targeting SPT with myriocin can counteract TGF-β–mediated signaling and RIPF progression, suggesting that SPT may be a novel therapeutic target for managing RIPF [36,101]. IR of the lung is associated with increased IL-4 expression and that of its receptor, IL4 receptor α1, as well as the dual oxidase 2 (DUOX2) gene. Treatment with metformin suppressed upregulation of IR-induced genes and IL-4, which was associated with amelioration of pathological changes. Treatment with metformin attenuated the upregulation of the IL-4–DUOX2 pathway and other pathological damages to the lung after exposure to a high dose of IR [102]. The heat shock protein 27 (HSP27) was also identified as a candidate target for treating PF in a mouse model of clinical SBRT. Inhibition of HSP27 using a recently identified small-molecule inhibitor, J2, induces crosslinking of HSP27 attenuated RIPF development. Mechanism underlying HSP27-mediated fibrosis development is IkBa-NFkB signaling activation, which is involved in the EMT process (Table 3) [103]. 

## 6. Conclusions

Tolerance of lung tissue to IR and subsequent development of IR pneumonitis and fibrosis limit the effectiveness of RT. However, despite the urgent need for improved treatment of RIPF, there are no clinically used methods for RIPF treatment. In addition to drug development, it is important to identify the predictive biomarker that can help identify patients susceptible to RIPF, so that a lower IR dose can be administered to these patients. Future research efforts should focus on identifying novel RIPF inhibitors to improve the treatment of patients with RIPF. Combining small animal irradiators with appropriate animal models and preclinical molecular and functional imaging studies has great potential to further enhance our understanding of the IR response of lung tissue. This approach may help address long-standing questions about the inter-relationships of acute and late effects of IR. This will further promote the development of effective novel treatments that can preserve lung function and quality of life after RT.

In this review, we emphasized the importance of small animal models that can mimic SBRT in terms of delivering high-dose focal irradiation. Regarding the level of vascular dysfunction in the lung tissue, it may occur after high-dose IR. Additional mechanisms are significantly affected in normal tissues after SBRT when compared to conventional RT. However, due to the limited understanding of basic mechanisms and the quantitative impact of clinical outcomes, many questions and investigations are needed to alleviate the effects of high-dose radiation on lung injury. Therefore, more examination using additional animal models are needed. 

In conclusion, recent advances have led to the development of more persistent experimental RIPF models and have allowed investigation of targeted epithelial damage, fibroblast-specific alterations, regulation of inflammatory cells during fibrosis, and epithelial-mesenchymal crosstalk. Although these models are still unable to replicate all the features of human RIPF pathology, specific analysis of signal pathways and interaction analysis among various cell types may be possible. More persistent models can enhance our ability to study mechanisms that are operative during the fibrogenic stage, which will increase the likelihood of translating the animal model findings to human disease and facilitate the assessment of therapeutic efficacy on fibrotic remodeling. This may enable a more precise prediction of which compounds have the ability to improve results after fibrosis is established.

## Figures and Tables

**Table 1:** Related cells in the development of radiation-induced pulmonary fibrosis (RIPF).

Classification		Characteristics	Reference
**Alveolar epithelial cells and vascular endothelial cells**		− Connection between these cells are damaged by IR ^1^, causing RIPF ^2^ due to dysregulation of myofibroblasts and excessive extracellular matrix (ECM) deposition	[58]
Fibroblast		− Its accumulation by IR is mediated by TGF-β1 ^3^, TGF-α1 ^4^, PDGF ^5^, CXCL12 ^6^ and other factors, of which the key molecular is TGF-β1	[59]
Macrophage	M1	− Existence predominantly within injured regions − Injury exacerbration and amplification of inflammatory response with pre-inflammatory cytokine release − Recruitment of fibroblasts and promote to myofibroblasts proliferation − Involvement of MMP (Matrix metalloproteinase) release that degrade ECM ^7^ and promotes EMT/ EndMT (Epithelial and endothelial-to-mesenchymal trasition)	[38,39,40,41,42,48,49]
	M2	− Involvement of an anti-inflammatory environment and wound healing promotion − Important pro-fibrotic role in the persistent injured lesion − Secretion of large amounts of profibrotic factors (TGF-β and galactin-3)	
Th1 and Th2 cells		− Important role in the development of RIPF − Th1 ^8^ mainly contributes to acute radiation pneumonitis, and Th2 mainly contributes to chronic RIPF.	[59]

^1^ IR: irradiation; ^2^ RIPF: radiation-induced pulmonary fibrosis; ^3^ TGF-β1:Transforming growth factor beta 1; ^4^ TGF-α1: Transforming growth factor alpha 1; ^5^ PDGF: Platelet-derived growth factor; ^6^ CXCL12: C-X-C motif chemokine ligand 12; ^7^ ECM: Extracellular matrix; ^8^ Th1: T helper type 1.

**Table 2:** Cytokines in the development of RIPF.

Name	Cells of Origin	Characteristics	Ref.
CCL2/MCP-1 ^1^	Macrophages, epithelium	− Monocyte chemo-attractant protein	[60]
		− Participates in the synthesis of proinflammatory cytokines	
CCL3/MCP-2 ^2^	Macrophages, epithelium	− Macrophage chemo-attractant protein	[29,60]
ET-1 ^3^	Endothelium, smooth muscle cells, fibroblasts	− Promotes vasoconstriction, stimulates ECM ^4^ production in fibroblasts	[61]
IFN-γ ^5^	Th1 cells (Type I helper cell)	− Involvement in inhibition of IL-4, IL-13, and TGF-β1-related pathways	[62]
		− Inhibition of Th2 cell differentiation and Th2-derived cytokines expression	
IL-1β ^6^	Monocytes, macrophages	− One of the strong pro-inflammatory cytokines	[63,64]
		− Induction and propagation of inflammation	
IL-13 ^7^	T cells (Thy-2 CD34+)	− Important pro-fibrotic cytokines and closely related with fibrosis of lung	[65,66]
		− Similar function to IL-4 (because of sharing the α chain of the IL-4 receptor)	
IL-4 ^8^	T cells (Thy-2 CD34+)	− Important role in the adaptive immune response	[67,68,69,70]
		− Increased expression of type I collagen, type III collagen, and fibronectin	
M-CSF ^9^	Multiple, including endothelium, epithelium, macrophages, platelets	− Major regulator involved in the survival, differentiation, and proliferation of monocytes/macrophages	[29]
		− Promotes to activate granulocytes	
PDGF ^10^	Multiple, including endothelium, epithelium, macrophages, platelets	− PDGFR-α ^11^ is related to fibrosis and transactivated by TGFβ1	[33,59,71]
		− Stimulation of the myofibroblast proliferation and the ECM synthesis via PDGF (platelet-derived growth factor) transduction major pathways	
TGF-β ^12^	Platelets, epithelium, endothelium, macrophages, fibroblasts, monocytes	− Converting fibroblast into matrix-producing myofibroblast	[33,58]
		− Promotes synthesis of collagen and inhibits synthesis of collagenase and plasminogen activator	
TNF-α ^13^	Activated T cells, macrophages	− Induction of NF-κB activity	[72,73,74]
		− Regulation of immunity and inflammatory gene expression	

^1^ CCL2 /MCP-1: C-C motif chemokine ligand 2/Monocyte chemoattractant protein 1; ^2^ CCL3/MCP-2: C-C motif chemokine ligand 3/Monocyte chemoattractant protein 2; ^3^ ET-1: Endothelin 1; ^4^ ECM: Extracellular matrix; ^5^ IFN-γ: Interferon gamma; ^6^ IL-1β: Interleukin 1 beta; ^7^ IL-13: Interleukin 13; ^8^ IL-4: Interleukin 4; ^9^ M-CSF: Macrophage colony stimulating factor; ^10^ PDGF: Platelet-derived growth factor; ^11^ PDGFR-α: Platelet-derived growth factor receptor-α; ^12^ TGF-β: Transforming growth factor- β; ^13^ TNF-α: Tumor necrosis factor- α.

**Table 3:** Therapeutic targets for radiation-induced pulmonary fibrosis (RIPF).

Name	Structure	Target	Mode of Action	Clinical Trials and NCT Number
Glucocorticosteroid		Inflammatory cells	− Main management in acute IR pneumonitis − Not clear in PF − Induce side effects on long-term use	
Sivelestat	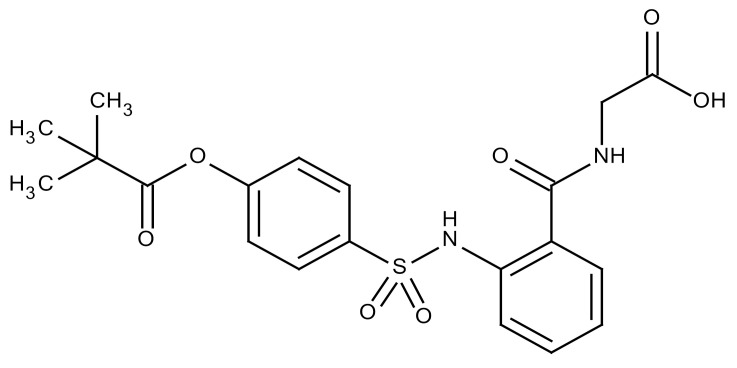	Neutrophil elastase	− Neutrophil elastase inhibitor − Decrease of collagen deposition and accumulation of neutrophils − Inhibition of RILI in mice − Inhibition of alveolitis and increased survival after IR	Phase 2, NCT00036062 (completed)
CS7		Anti-CSF1R	− Anti-CSF1R antibody − Depletion of IM in RIPF model→Reverse PF − Murine counterpart of the human CS4 antibody	Research
ABT-263 (Navitoclax)	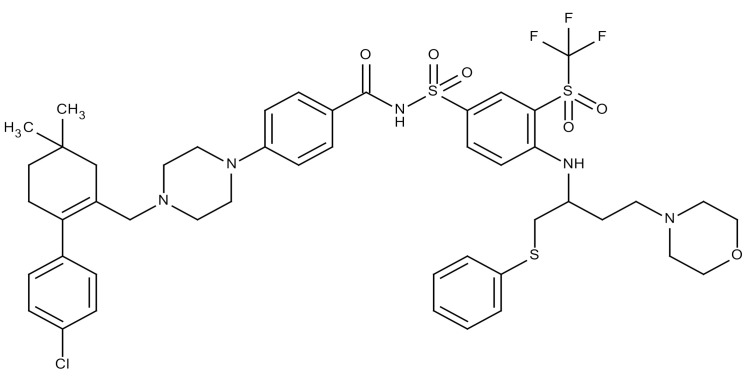	Bcl-2/xl	− A specific Bcl-2/xl inhibitor and a senolytic drug − Reverse PF after thoracic IR in mice model	Research
BBT-877		Autotaxin (ATX)	− Autotaxin inhibitor	Phase 1, NCT03830125 (completed)
GLPG1690	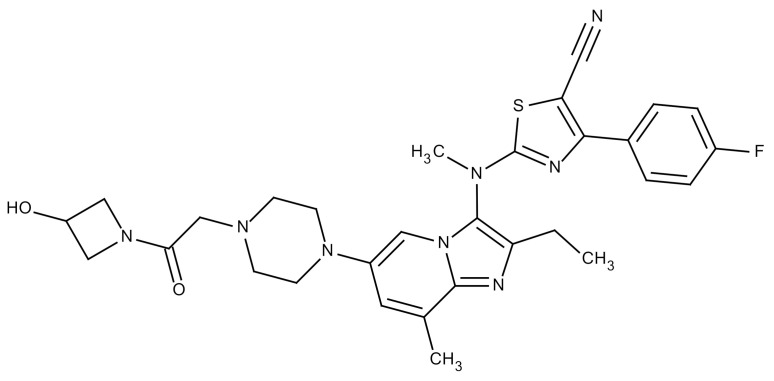	Autotaxin (ATX)	− Autotaxin inhibitor	Phase 2, NCT02738801 (completed)
GLPG1205	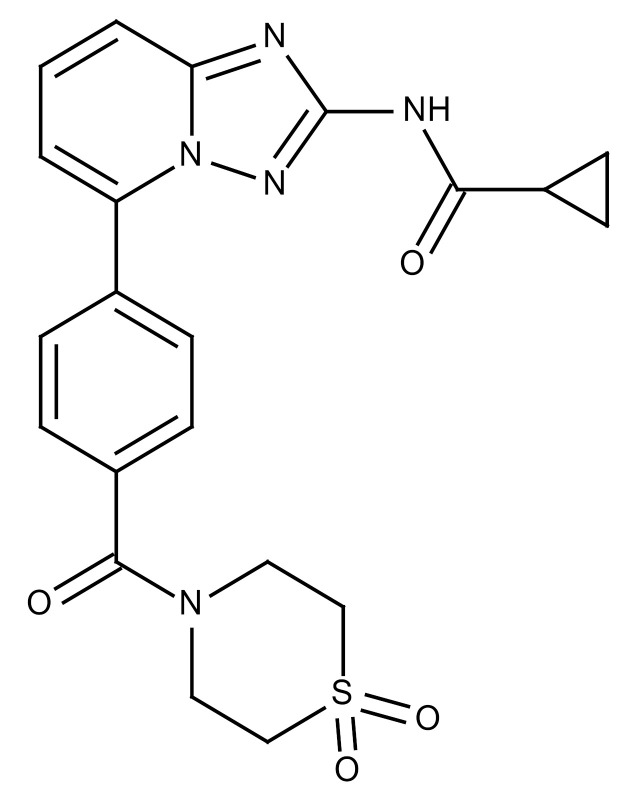	GPR84	− Specific GPR84 inhibitor	Phase 2, NCT03725852 (Active)
PBI-4050	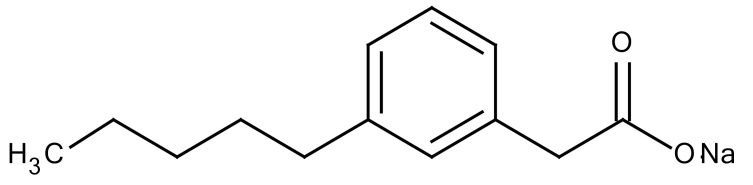	GPR40 and GPR84	− Activation of GPR40 receptor	Phase 2, NCT02538536 (completed)
			− Suppression of GPR84 activity	
BMS-986020	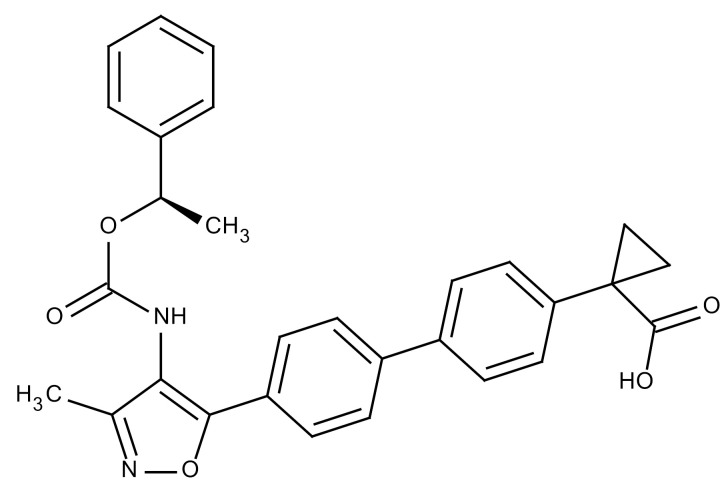	Lysophosphatidic acid (LPA) receptor	− Selective inhibition of the LPA receptor 1	Phase 2, NCT01766817 (completed)
Ifenprodil (NP-120)	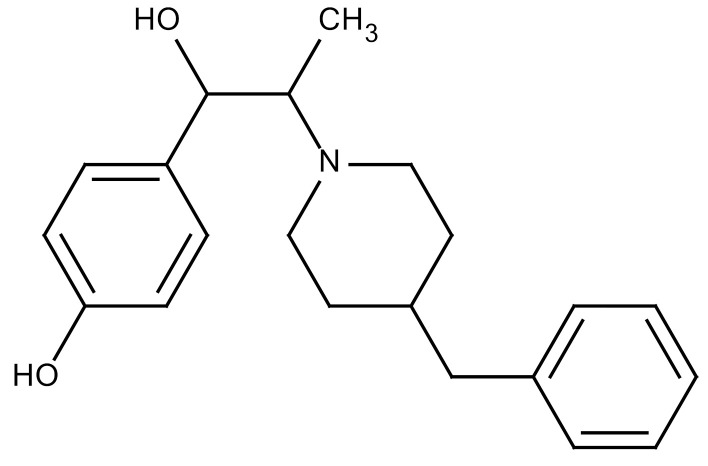	NDMA receptor	− Repurposing of circulatory drug − N-methyl-d-aspartate (NDMA) receptor glutamate receptor antagonists	Phase 3, NCT02722304 (terminated)
PXS-5382A		LOXL2	− LOXL2 inhibitor	Research
PAT-1251	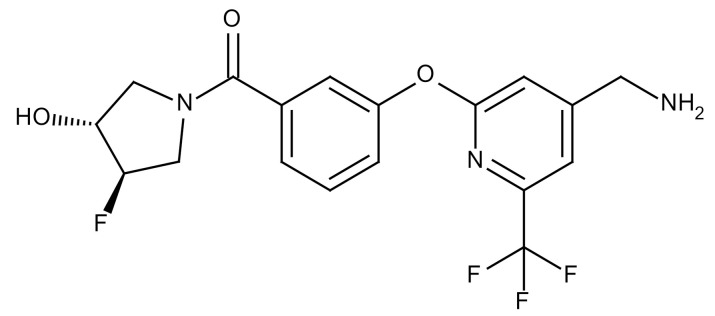	LOXL2	− LOXL2 inhibitor	Research
KD025	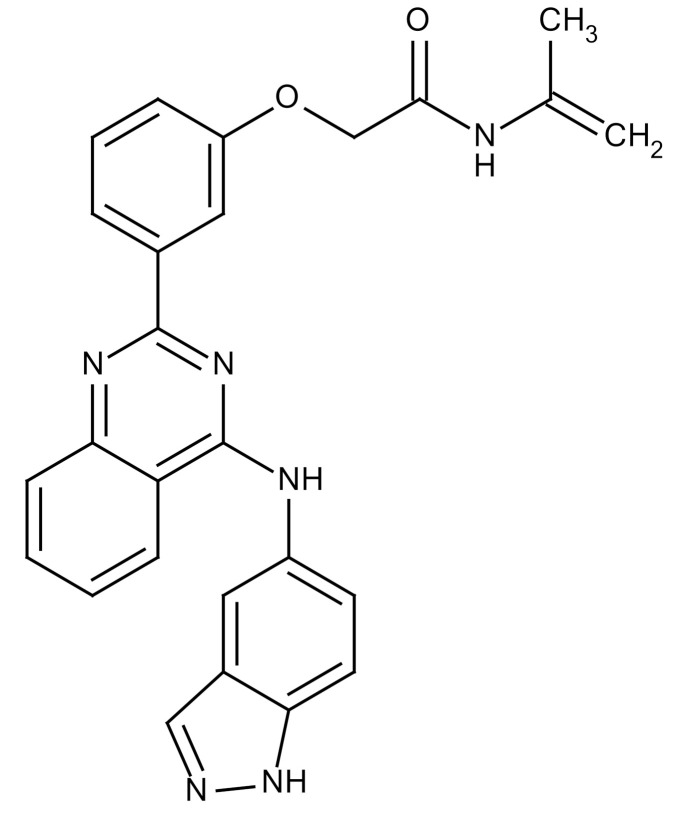	Rho-associated coiled-coil kinase or ROCK2	− Inhibition of Rho-associated coiled-coil kinase or ROCK2	Phase 2, NCT02688647(active)
BLD-2660		Calpain	− Calpain inhibitor	Phase 2, NCT04244825 (Not yet recruiting)
Pamrevlumab	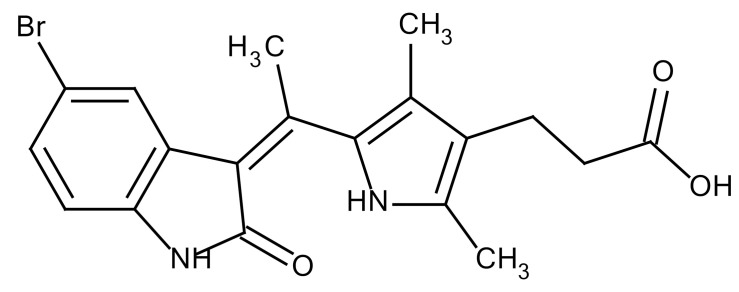	CTGF (Connective Tissue Growth Factor)	− Recombinant human monoclonal antibody − CTGF inhibitor − Restoring lung function in the mouse model − Inhibition of M2 polarized macrophage influx and myofibroblast abundance − Normalizing IR-induced gene expression changes	Phase 3, NCT03955146 (recruiting)
SU9518		PDGF	− PDGFR tyrosine kinase inhibitor − Inhibition of collagen deposition, alveolar wall thickness, and other histologically visible signs of fibrosis in a mouse model − Inhibition of IR-induced fibroblast and endothelial cell activation	Research
SU11657		PDGF	− PDGFR tyrosine kinase inhibitor	Research
Imatinib	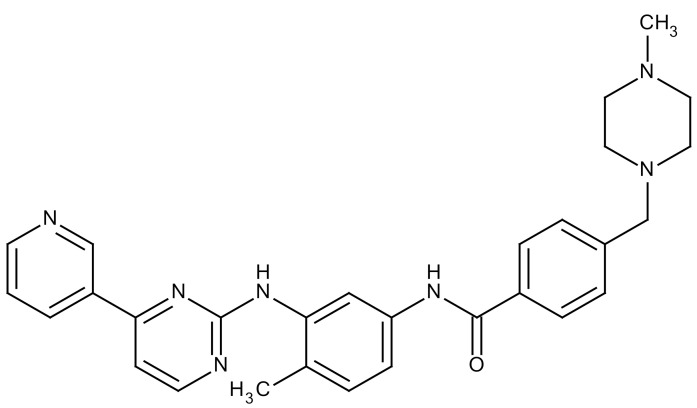	PDGF	− Inhibitor of tyrosine kinases of the TGFβ and PDGF pathways − Inhibition of RIPF in a mouse model	Phase 1, NCT03328117 (completed)
Pirfenidone	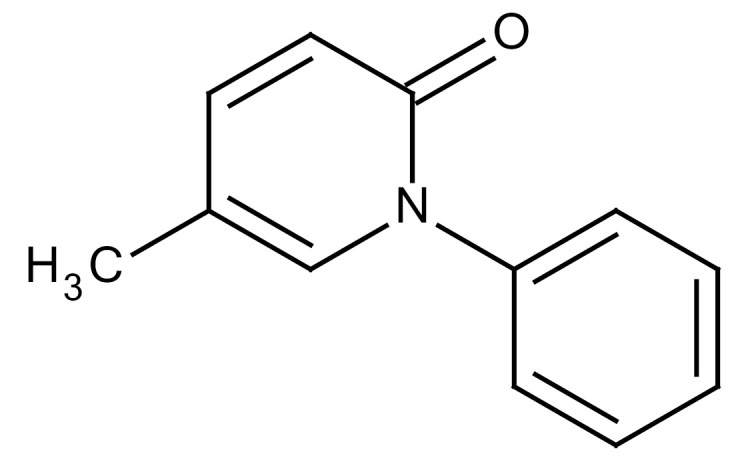	Multiple targets	− Inhibition of TGF-β, TNF, IL-10, p38α and p38γ, and p38α	Clinical use
Thalidomide	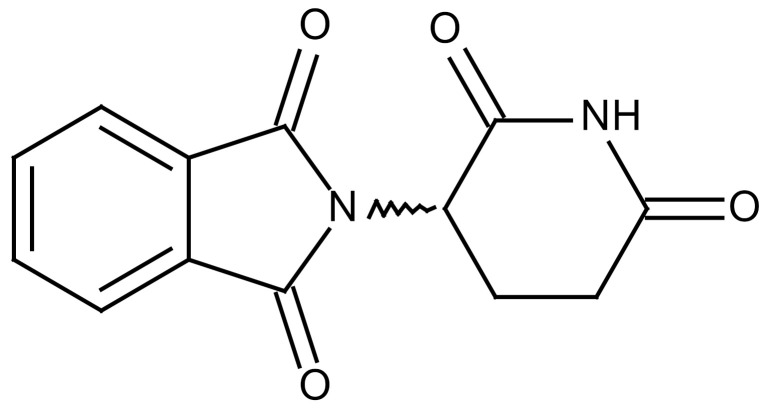	TGF-β1	− Inhibition of TGF-β1-induced α-SMA and vimentin production and TGF-β1-induced cell morphological changes by suppressing both Smad-dependent and non-Smad-dependent pathways.	Phase 3, NCT00600028 (completed)
SM16	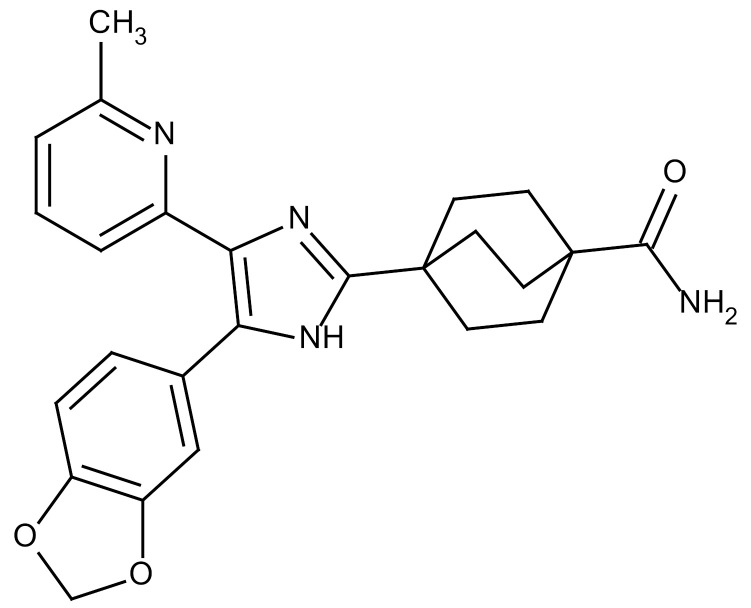	TGF-β RI	− TGF-β RI inhibitor	Research
LY2109761	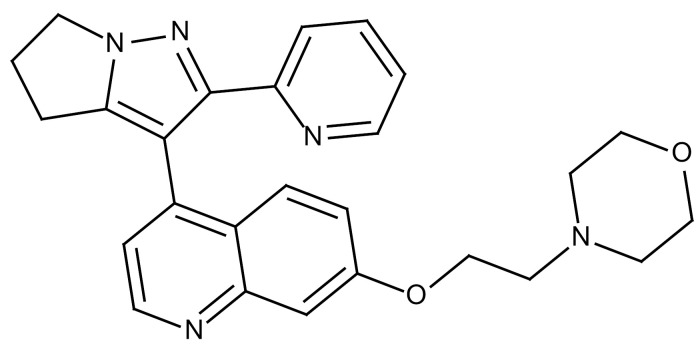	TGF-βRI/II	− Dual inhibitor of TGF-β RI/II	Research
Galunisertib	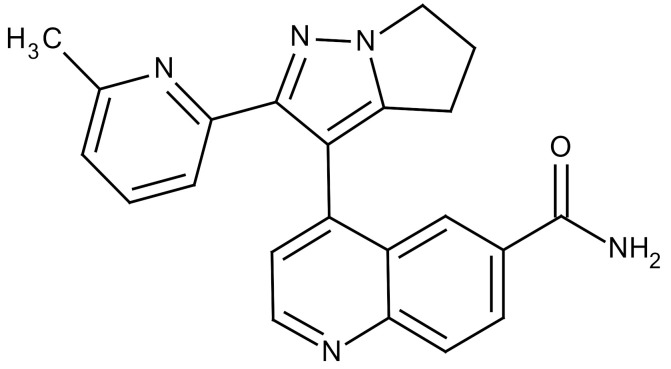	TGF-β RI	− Highly selective TGF-β RI inhibitor	Research
SB203580	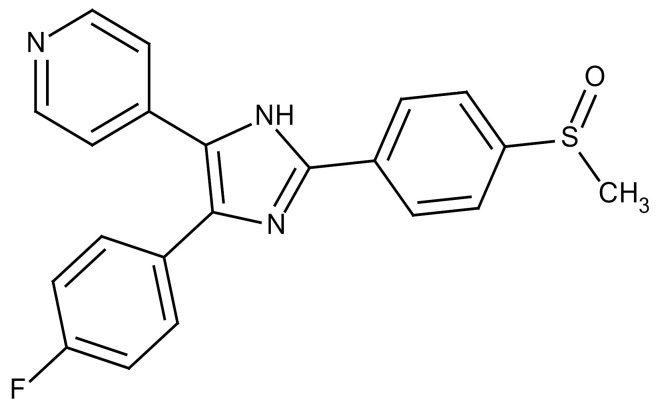	TGF-β RI	− TGF-β RIkinase inhibitor − Inhibitor of TGF-β/Smad signal transduction	Research
WP631	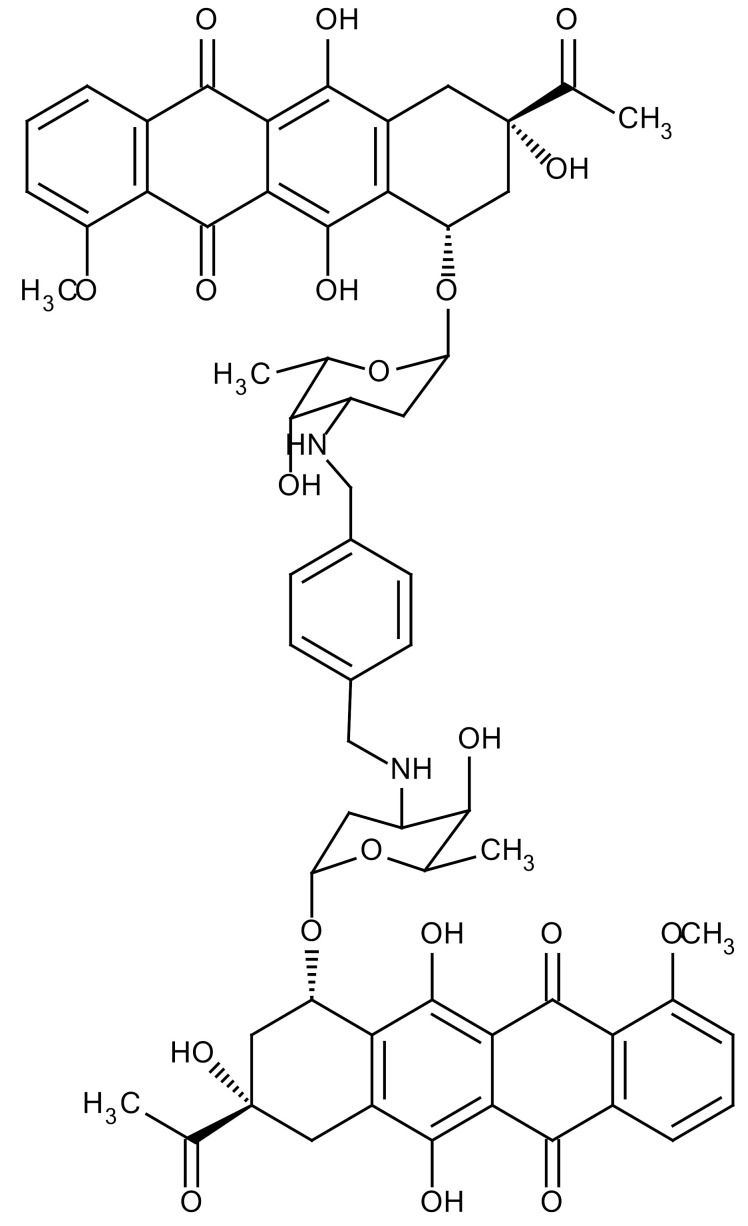	TGF-β/Smad	− DNA intercalator − Inhibitor of TGF-β/Smad signal transduction − Inhibition of PAI-1	Research
MyD88		Recombinant protein	− A key intracellular adaptor of TLR signaling − Involving innate immunity and NF-κB activation responses − Absence of MyD88 results in unresolved pulmonary infiltrates and enhanced collagen deposition	Research
MSX-122	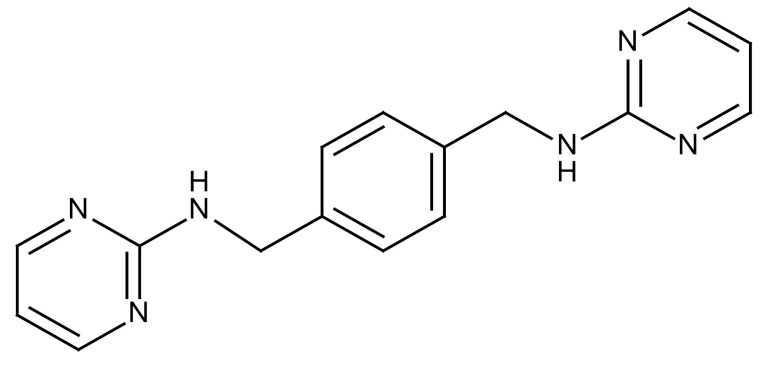	CXCR4	− A novel small molecule and partial CXCR4 antagonist − CXCR4/CXCL12-axis is critical in the development of RIPF in a mouse model − Reduces RIPF	Research
PLN-74809		Integrin αvβ6, αvβ1	− Selective inhibitor of the αvβ6 and αvβ1 integrins	Phase 2, NCT04072315 (recruiting)
IDL-2965		Integrin αvβ1, αvβ3, and αvβ6	− Inhibitor of three integrin αvβ1, αvβ3, and αvβ6	Phase 1, NCT03949530 (recruiting)
			− Blocks the activation of TGF-β	
PRM-151		Amyloid P/Pentraxin 2 protein	− A recombinant human serum amyloid P/Pentraxin 2 protein	Phase 2, NCT02550873 (active)
Omipalisib (GSK2126458)	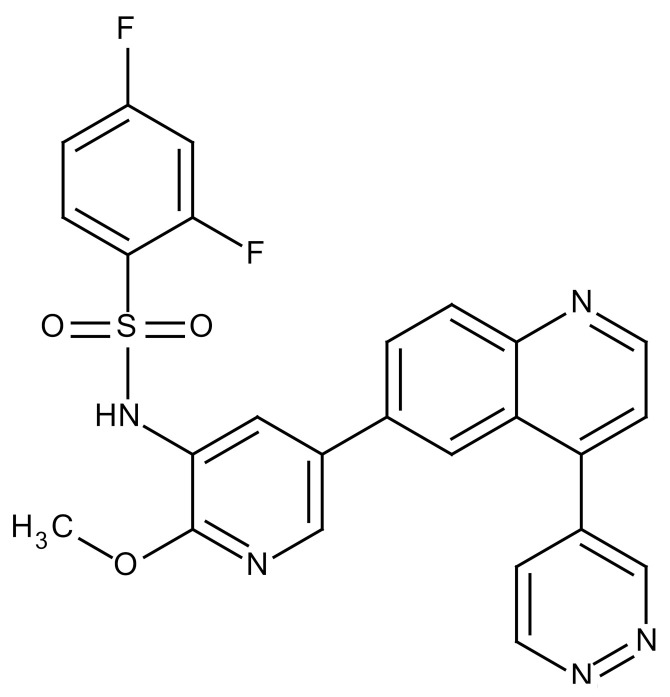	PI3K and mTOR signals	− Inhibitor of PI3K and mTOR signals	Phase 1, NCT01725139 (completed)
Nintedanib	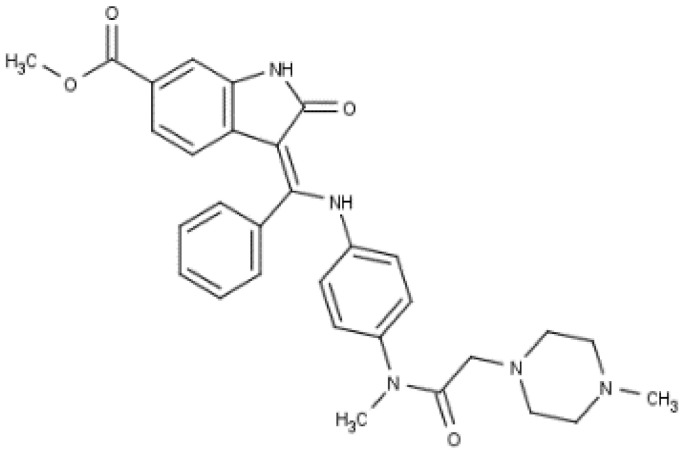			Clinical use
Amifostine	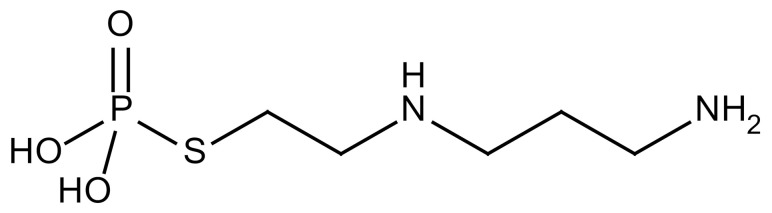	Reactive oxygen species (ROS)	− Active metabolite scavenger of ROS − Radioprotector approved by FDA − Inhibition of plasma level of TGF-β − Prevents accumulation of macrophage and expression of TGF-β	Research
SOD-TAT		ROS (Recombinant protein)	− Pretreatment enhances antioxidant ability and reduced RIPF in mice	Research
2-ME (2-Methoxy-estradiol)	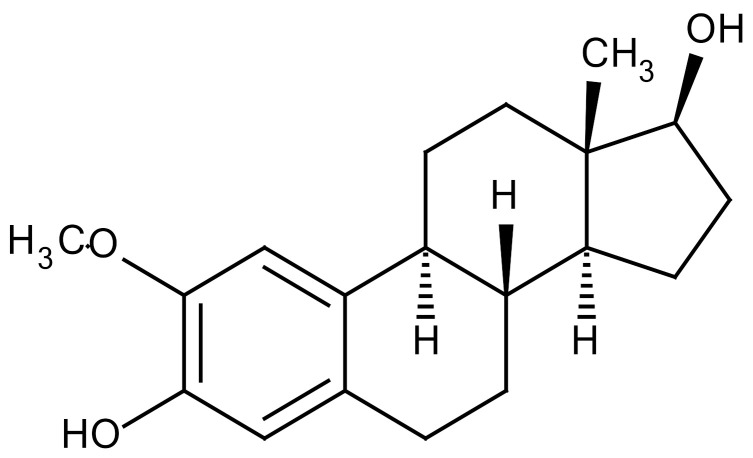	HIF-α (Natural metabolite of estradiol)	− Inhibition of HIF-α − Inhibition of EndMT and deposition of vascular collagen	Research
Myriocin	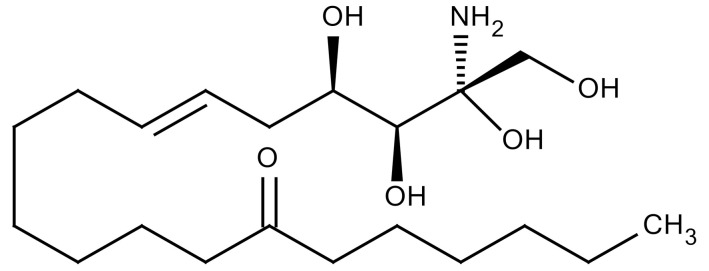	SPT	− Inhibitor of serine palmitoyl transferase (SPT) − Inhibition of de novo biosynthesis of sphingolipids	Research
Metformin	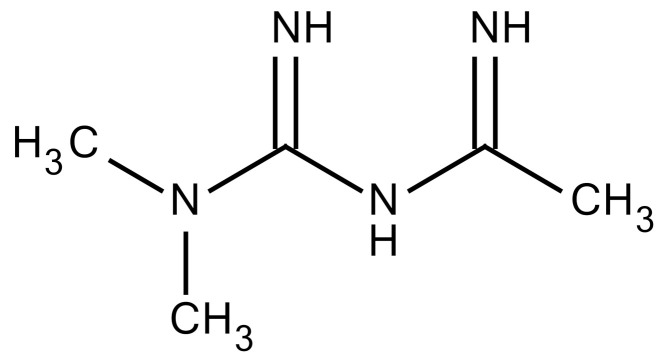		− Inhibition of IL-4-DUOX2 pathway − Inhibition of infiltration of macrophages and lymphocytes	Research
ACE inhibitors			− Inhibition of morbidity during the pneumonitis phase − Inhibition of RIPF in the mouse model	Research

Abbreviations: IR: Irradiation; PF: Pulmonary fibrosis; RILI: Radiation-induced lung injury; IM: Interstitial macrophage; PDGFR: Platelet-derived growth factor receptor; IL: Interleukin; TGF-β: Transforming growth factor-β; TNF: Tumor necrosis factor; PAI-1: Plasminogen Activator inhibitor-1; TLR: Toll-like receptor; SOD-TAT: Superoxide dismutase fusion of TAT; ROS: Reactive oxygen species; HIF-1α: Hypoxia-inducible factor 1 alpha.* Clinical trials and NCT number: https://www.clinicaltrials.gov/ct2/home.
